# Maternal inheritance of bifidobacterial communities and bifidophages in infants through vertical transmission

**DOI:** 10.1186/s40168-017-0282-6

**Published:** 2017-06-26

**Authors:** Sabrina Duranti, Gabriele Andrea Lugli, Leonardo Mancabelli, Federica Armanini, Francesca Turroni, Kieran James, Pamela Ferretti, Valentina Gorfer, Chiara Ferrario, Christian Milani, Marta Mangifesta, Rosaria Anzalone, Moreno Zolfo, Alice Viappiani, Edoardo Pasolli, Ilaria Bariletti, Rosarita Canto, Rosanna Clementi, Marina Cologna, Tiziana Crifò, Giuseppina Cusumano, Sabina Fedi, Stefania Gottardi, Claudia Innamorati, Caterina Masè, Daniela Postai, Daniela Savoi, Massimo Soffiati, Saverio Tateo, Anna Pedrotti, Nicola Segata, Douwe van Sinderen, Marco Ventura

**Affiliations:** 1 Laboratory of Probiogenomics, Department of Chemistry, Life Sciences and Environmental Sustainability, University of Parma, Parco Area delle Scienze 11a, 43124 Parma, Italy; 2 Centre for Integrative Biology, University of Trento, Trento, Italy; 3 APC Microbiome Institute and School of Microbiology, Bioscience Institute, National University of Ireland, Cork, Ireland; 4 Azienda Provinciale per i Servizi Sanitari, Trento, Italy; 5 GenProbio srl, Parma, Italy

**Keywords:** Microbiota, Virome, Microbiome, Bifidobacteria, Vertical transmission

## Abstract

**Background:**

The correct establishment of the human gut microbiota represents a crucial development that commences at birth. Different hypotheses propose that the infant gut microbiota is derived from, among other sources, the mother’s fecal/vaginal microbiota and human milk.

**Results:**

The composition of bifidobacterial communities of 25 mother-infant pairs was investigated based on an internal transcribed spacer (ITS) approach, combined with cultivation-mediated and genomic analyses. We identified bifidobacterial strains/communities that are shared between mothers and their corresponding newborns. Notably, genomic analyses together with growth profiling assays revealed that bifidobacterial strains that had been isolated from human milk are genetically adapted to utilize human milk glycans. In addition, we identified particular bacteriophages specific of bifidobacterial species that are common in the viromes of mother and corresponding child.

**Conclusions:**

This study highlights the transmission of bifidobacterial communities from the mother to her child and implies human milk as a potential vehicle to facilitate this acquisition. Furthermore, these data represent the first example of maternal inheritance of bifidobacterial phages, also known as bifidophages in infants following a vertical transmission route.

**Electronic supplementary material:**

The online version of this article (doi:10.1186/s40168-017-0282-6) contains supplementary material, which is available to authorized users.

## Background

The period immediately following birth is believed to be crucial for the correct establishment of the gut microbiota with possible temporary and long-lasting effects on host health [1]. During this infant stage of (human) life, microorganisms originating from the mother and from environmental microbial communities rapidly colonize the gastrointestinal tract (GIT) of the neonate to form the early infant microbiota whose establishment is also influenced by the feeding method [2–7]. Bifidobacteria are among the first colonizers of the intestine of newborns and are considered to play pivotal roles in terms of modulation of mucosal physiology and innate immunity of the host [6, 8, 9]. Genomically identical bifidobacterial strains have been isolated from fecal samples of mother and child combinations, as well as the corresponding human milk samples, indicative of a vertical transmission route from maternal GIT to (breastfed) infants [10–13]. This has given rise to the hypothesis that microbial colonization of the infant depends on the mother’s fecal/vaginal microbiota as well as on (providing) breast milk [14]. Bacteria may reach the mammary gland of the mother and may be transmitted directly to breastfed infants, although, inversely, bifidobacteria may be introduced into human milk from the infant’s oral cavity during suckling [14–17].

Notably, several studies have highlighted the possibility of vertical transmission of (components of) the gut microbiota from mother to child [12, 15, 18]. Furthermore, a recent pilot study involving four mother-child pairs revealed direct transmission of bifidobacterial strains from mothers to their newborns using a novel ITS-based approach [11]. Although intriguing, the biological relevance of such findings in this latter work is limited due to the small number of mother-infant pairs analysed [11].

Another important component of the gut microbiota is represented by virus-like particles in the GIT, which together constitute the gut virome [19, 20]. Currently, very little is known about the infant virome [21–23], although the existence and impact of phages specific for bifidobacteria, i.e., bifidophages, in the infant gut has recently been discribed [24].

In the current study, we analyzed the composition of the bifidobacterial communities of 25 mother-infant pairs by means of the ITS profiling approach mentioned above, combined with cultivation and genome investigations. Employing a next-generation sequencing (NGS) approach, we identified bifidobacterial strains that are shared between mothers and their corresponding newborns, while we furthermore obtained evidence for vertical, mother-child transmission of bifido(pro)phages.

## Methods

### Subject recruitment and sample collection

The study protocol was approved by the Ethical Committee of the “Azienda Provinciale per i Servizi Sanitari” in Trento, Italy, as well as by the Ethical Committee of the University of Parma, Italy, and informed written consent was obtained from all participants or their legal guardians. Twenty-five mother-infant pairs were enrolled in this study, which were selected based on the following criteria: the mother’s age (ranging in age from 18 to 40 years old) and those subjects who were not taking any probiotics or antibiotics. All subjects were considered to be healthy, as based on self-reporting. At the time of delivery, during birth or immediately after it, stool samples were collected of 21 mothers. Stool samples of infants were collected at two different time points, i.e., 7 days and 1 month following birth of the infant. Furthermore, 10 ml of breast milk sample of each mother was collected in sterile tubes (Table 1). For the sample set TVPR_03, we collected stool samples of infants and breast milk samples at 1 month following birth. Stool and milk samples were collected following the “Stool Packaging Instructions” of the “Core Microbiome Sampling Protocol A” reported in the “Manual of Procedures of the Human Microbiome Project” (http://www.hmpdacc.org/doc/HMP_MOP_Version12_0_072910.pdf) [25]. Samples were immediately frozen at −20 °C and shipped under frozen conditions to the laboratory, where they were immediately processed. DNA extractions of stool samples and human milk samples were performed as previously described [11].

**Table 1:** List of samples included in this study

CoupleID	Samples	
TVTR_10005	Mum-T0	2062
	Milk-T3	2072
	Infant-T3	2074
	Milk-T4	2076
	Infant-T4	2078
TVTR_10006	Milk-T3	2090
	Infant-T3	2092
	Milk-T4	2094
	Infant-T4	2096
TVTR_10007	Milk-T3	2108
	Infant-T3	2110
	Milk-T4	2112
	Infant-T4	2114
TVTR_10008	Milk-T3	2126
	Infant-T3	2128
	Milk-T4	2130
	Infant-T4	2132
TVTR_10009	Mum-T0	2134
	Milk-T3	2144
	Infant-T3	2146
	Milk-T4	2148
	Infant-T4	2150
TVTR_10010	Mum-T0	2152
	Milk-T3	2162
	Infant-T3	2164
	Milk-T4	2166
	Infant-T4	2168
TVTR_10017	Mum-T0	2278
	Milk-T3	2287
	Infant-T3	2290
	Milk-T4	2292
	Infant-T4	2293
TVTR_10019	Mum-T0	2314
	Milk-T3	2324
	Infant-T3	2326
	Milk-T4	2328
	Infant-T4	2330
TVTR_10020	Mum-T0	2332
	Milk-T3	2342
	Infant-T3	2344
	Milk-T4	2346
	Infant-T4	2347
TVTR_10021	Mum-T0	2350
	Milk-T3	2360
	Infant-T3	2362
	Milk-T4	2364
	Infant-T4	2365
TVTR_10023	Milk-T3	2396
	Infant-T3	2398
	Milk-T4	2400
	Infant-T4	2401
TVTR_10024	Mum-T0	2404
	Milk-T3	2414
	Infant-T3	2416
	Milk-T4	2418
	Infant-T4	2419
TVTR_10025	Mum-T0	2422
	Milk-T3	2432
	Infant-T3	2434
	Milk-T4	2436
	Infant-T4	2437
TVTR_10028	Mum-T0	2476
	Milk-T3	2486
	Infant-T3	2487
	Milk-T4	2490
	Infant-T4	2491
TVTR_10029	Mum-T0	2492
	Milk-T3	2502
	Infant-T3	2504
	Milk-T4	2506
	Infant-T4	2508
TVTR_10030	Mum-T0	2509
	Milk-T3	2519
	Infant-T3	2521
	Milk-T4	2523
	Infant-T4	2525
TVTR_10031	Mum-T0	2526
	Milk-T3	2536
	Infant-T3	2538
	Milk-T4	2540
	Infant-T4	2541
TVTR_10032	Mum-T0	2544
	Milk-T3	2554
	Infant-T3	2556
	Milk-T4	2558
	Infant-T4	2559
TVTR_10034	Mum-T0	2580
	Milk-T3	2590
	Infant-T3	2592
	Milk-T4	2594
	Infant-T4	2595
TVTR_10035	Mum-T0	2598
	Milk-T3	2608
	Infant-T3	2610
	Milk-T4	2612
	Infant-T4	2613
TVTR_10036	Mum-T0	2616
	Milk-T3	2626
	Infant-T3	2628
	Milk-T4	2630
	Infant-T4	2632
TVTR_10038	Mum-T0	2652
	Milk-T3	2662
	Infant-T3	2664
	Milk-T4	2666
	Infant-T4	2668
TVPR_01	Mum-T0	3000
	Milk-T4	3001
	Infant-T4	3002
TVPR_02	Mum-T0	3006
	Milk-T3	3007
	Infant-T3	3008
	Milk-T4	3009
	Infant-T4	3010
TVPR_03	Mum-T0	3011
	Milk-T4	3012
	Infant-T4	3013

### ITS gene amplification and MiSeq sequencing

Partial ITS sequences were amplified from extracted DNA using the primer pair Probio-bif_Uni/Probio-bif_Rev, which targets the spacer region between the 16S rRNA and the 23S rRNA genes within the ribosomal RNA (rRNA) locus [26]. Illumina adapter overhang nucleotide sequence was then added to the generated ITS amplicons of *c.* 200 bp. The library of ITS amplicons was prepared following the 16S Metagenomic Sequencing Library Preparation Protocol (Part No. 15044223 Rev. B—Illumina). Sequencing was performed using an Illumina MiSeq sequencer with MiSeq Reagent Kit v3 chemicals.

### ITS microbial profiling analysis

Profiling of known bifidobacterial species was performed using the primer pair Probio_bif_uni/Probio_bif_rev, an improved bifidobacterial ITS database encompassing all publicly available bifidobacterial genomes and a custom bioinformatics script, as described previously [26].

### Evaluation of the bifidobacterial cell density by qPCR

The presence of bifidobacteria in infant stool samples was evaluated using quantitative real-time PCR (qRT-PCR). The primers used in this study are Probio_bif_uni/Probio_bif_rev to determine numbers of bifidobacteria [26] and Probio_uni/Probio_rev to quantify total bacterial numbers [27]. The quantitative contribution of bifidobacteria to the overall infant gut microbiota of each sample was evaluated by a ratio of the genome copy number ITS gene/16S rRNA gene (the genes targeted were in the same copy per genome). qPCR was performed using SoFast EvaGreen Supermix (Bio-Rad) on a CFX96 system (BioRad, CA, USA) following previously described protocols [28].

### Recovery of bifidobacteria on selective media

One gram of each fecal sample or 1 ml of human milk was mixed with 9 ml of phosphate-buffered saline (PBS), pH 6.5. Serial dilutions and subsequent plating were performed using the de Man-Rogosa-Sharpe (MRS) agar (Scharlau Chemie, Barcelona, Spain), supplemented with 0.05% (wt/col) l-cysteine hydrochloride and 50 μg/ml mupirocin (Delchimica, Italy). The agar plates were incubated in an anaerobic atmosphere (2.99% H_2_, 17.01% CO_2_, and 80% N_2_) in a chamber (Concept 400; Ruskin) at 37 °C for 48 h. Ten colonies were taken as a representation of the bacterial strains retrieved from the selective medium. DNA was extracted and subjected to (sub)-species identification as previously described [11].

### De novo genome sequencing and bioinformatics analyses

The genome sequence of the new bifidobacterial isolates was determined by GenProbio srl (Parma, Italy) using a MiSeq platform (Illumina, UK). A genome library was generated following the TruSeq Nano DNA library Prep protocol (Part No. 15041110 Rev. D). Library samples were loaded into a Flow Cell V3 600 cycles (Illumina) according to the technical support guide, and generated reads were depleted of adapter sequences, quality-filtered, assembled and protein-encoding open reading frame (ORF) predicted through the MEGAnnotator pipeline [29].

### Fermentation profiles on milk glycans

Prior to carrying out growth profile assays, *Bifidobacterium* cultures were grown from stock in Difco MRS (BD) and sub-cultured twice in the same medium. Subsequently, 5 ml of freshly prepared modified MRS (mMRS) medium [peptone from casein, 10 g/L; meat extract, 10 g/L, and yeast extract, 5 g/L (purchased from Difco); K_2_HPO_4_, 3 g/L; KH_2_PO_4_, 3 g/L; pyruvic acid, 0.2 g/L; polysorbate 80, 1 mL/L; tri-ammonium citrate, 2 g/L; MgSO_4_·7H_2_O, 0.575 g/L; MnSO_4_·4H_2_O, 0.12 g/L; cysteine-HCl, 0.3 g/L; and FeSO_4_·7H_2_O, 0.034 g/L], supplemented with 0.05% cysteine hydrochloride, and 1% of a particular carbohydrate was inoculated with 50 μl (1%) of a stationary phase culture. All carbohydrates (fucose, lactose, sialic acid, 3′-sialyllactose, 6′-sialyllactose, 2′-fucosyllactose, 3′-fucosyllactose, lacto-*N*-tetraose, or lacto-*N*-neotetraose) were dissolved in water and then sterilized by filtration (0.2-μ filter size) and then added to mMRS after sterilization by autoclaving. Uninoculated mMRS medium was used as a negative control. Cultures were incubated under anaerobic conditions in a modular atmosphere-controlled system (Davidson and Hardy, Belfast, Ireland) at 37 °C for 24 h, and the optical density at 600 nm (OD600) was determined manually, using a Shimadzu UV-1280 UV-399 VIS Spectrophotometer. Growth assays were carried out in triplicate, with standard deviation calculated for error values.

### Shotgun metagenomic analyses

Fastq files obtained from metagenomic sequencing of infant and mother fecal samples, as well as milk samples, were used as input for the SPAdes assembler (version 3.9, using default parameters and enabling the metagenomic option—meta) for de novo metagenomic assemblies [30]. ORFs were predicted with Prodigal [31] and annotated using NCBI RefSeq databases through RAPSearch2 aligner (cut-off *E* value of 1 × 10^−30^) [32]. Each contig was taxonomically classified by means of the gene hit obtained through the NCBI RefSeq databases by the use of the Contig Classifier (CoCla) script [33, 34]. Species-specific contigs were subdivided in different files, obtaining reconstructed genomes of the most abundant (bifido)bacterial taxa within each analysed sample.

### Bifidophage analyses

Contigs attributed to bifidobacterial (sub)species, by means of the CoCla script, were screened for the presence of bifidobacterial phages/prophages using a custom database composed of previously identified bifidophages [24]. Identified phage sequences were further manually curated in order to unveil overlapping extremities within the contig sequences. The presence of putatively identified bifidophages was screened in mother and infant fecal samples, and milk samples by PCR based on unique genes of that particular bifidophage.

## Results and discussion

### Evaluation of the bifidobacterial composition of the gut microbiota of infants

**Fig. 1:**
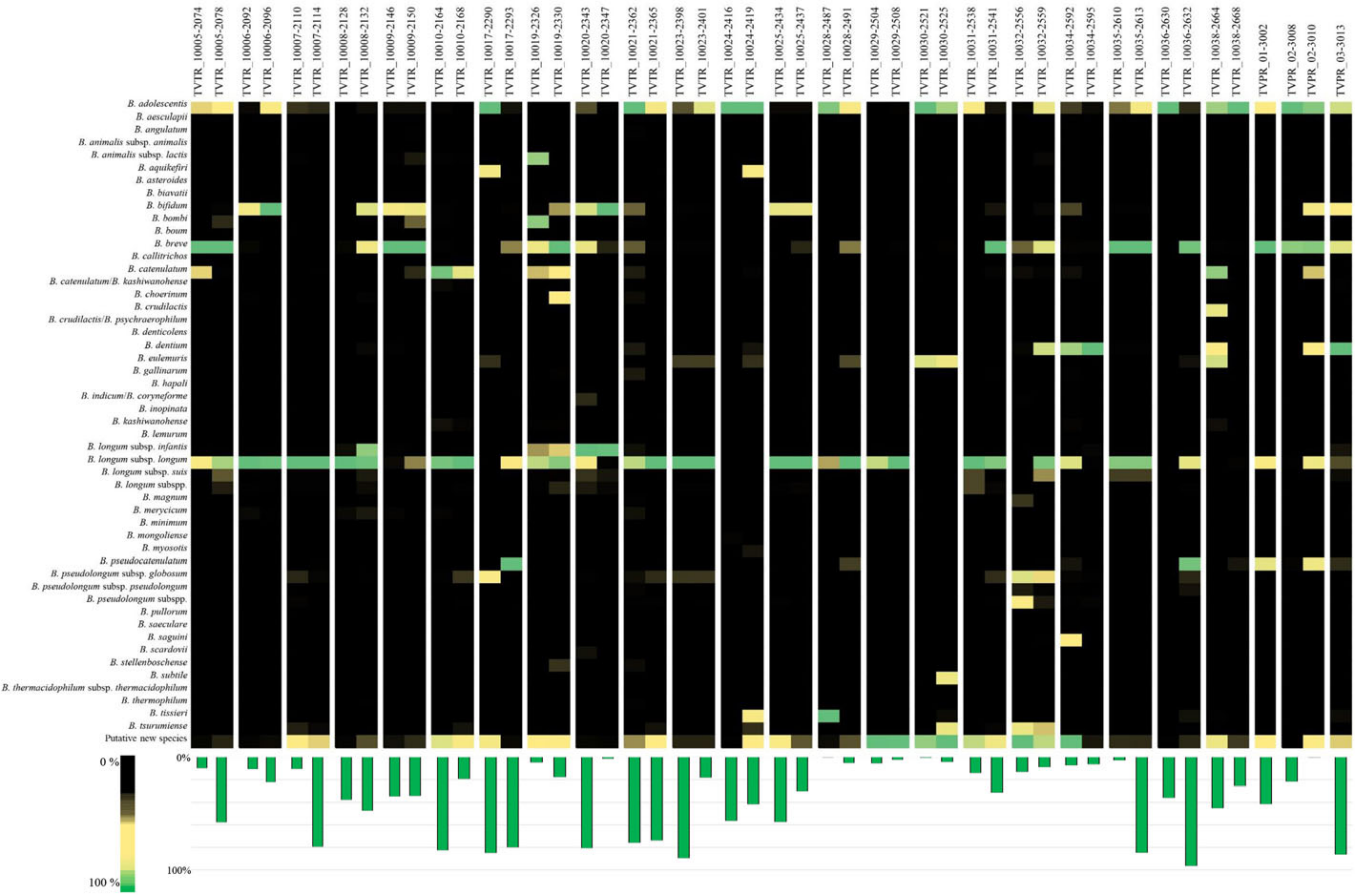
Profiling of the bifidobacterial communities of infant fecal samples. The heat map representation shows the relative abundance of *Bifidobacterium* species. On the bottom of the image, the *bar plots* show qPCR data regarding the proportion (in %) of bifidobacteria relative to other bacteria present in a given sample

To characterize the bifidobacterial population in the infant gut, we employed a recently developed pipeline based on sequencing of a hypervariable ITS region, here referred to as ITS profiling [26]. We collected fecal samples of 25 infants at two different time points, i.e., 7 days and 1 month following birth (Table 1). ITS profiling of these 50 samples produced a total of 1,703,642 reads, ranging from 1224 to 113,414 reads per sample (Additional file 1: Table S1), which were grouped into clusters of identical sequences (OTUs or operational taxonomic units) and then taxonomically classified. OTU analysis revealed that the dominant bifidobacterial species detected in the investigated fecal samples were *Bifidobacterium longum* subsp. *longum*, *Bifidobacterium breve*, *Bifidobacterium bifidum*, *Bifidobacterium longum* subsp. *infantis*, *Bifidobacterium adolescentis*, and *Bifidobacterium pseudocatenulatum*. In addition, ITS-based OTUs display a similarity level below 93% to any of the ITS sequences of the current known bifidobacterial (sub)species (Fig. 1 and Additional file 2: Figure S1). As previously discussed by Milani et al. [26], the comparison between bifidobacterial-type strain ITS sequences reveal that different bifidobacterial (sub)species possess ITS identity above 98%; it is therefore a distinct possibility that identified ITS-based OTUs with a similarity level below 93% represent novel bifidobacterial (sub)species. Notably, and in contrast to what currently is known about the composition of the bifidobacterial communities of the infant gut, we identified that such populations are composed not only by typical infant-gut bifidobacterial species, such as *B. breve*, *B. bifidum*, and *B. pseudocatenulatum*, but also by adult-associated bifidobacterial taxa, like *B. adolescentis* and *B. catenulatum* [11, 15]. This finding suggests that an ecological division based on age is not valid or at least not absolute, thus underscoring a possible strain-sharing scenario between adult and infant microbiotas. Furthermore, in seven and six cases, we observed a high abundance of non-common infant gut bifidobacteria such as *Bifidobacterium tissieri* and *Bifidobacterium dentium*, respectively (Fig. 1 and Additional file 2: Figure S1)*.* In order to assess the quantitative contribution of bifidobacteria to the overall infant gut microbiota of each sample, the total bacterial load as well as the bifidobacterial cell count were evaluated by qRT-PCR. These analyses indicate that bifidobacteria represent between 0.05 and 96.44% of the overall infant gut microbiota (average of 36.54%), ranging from 2.48E + 03 to 8.22E + 08 genome copy number per gram of feces. No significant differences between the two time points were detected. These findings strengthen the now well-established notion that bifidobacteria represent one of the dominant genera in the infant gut environment (Fig. 1) [10, 35–39].

### Sharing of OTUs between mothers and children

**Fig. 2:**
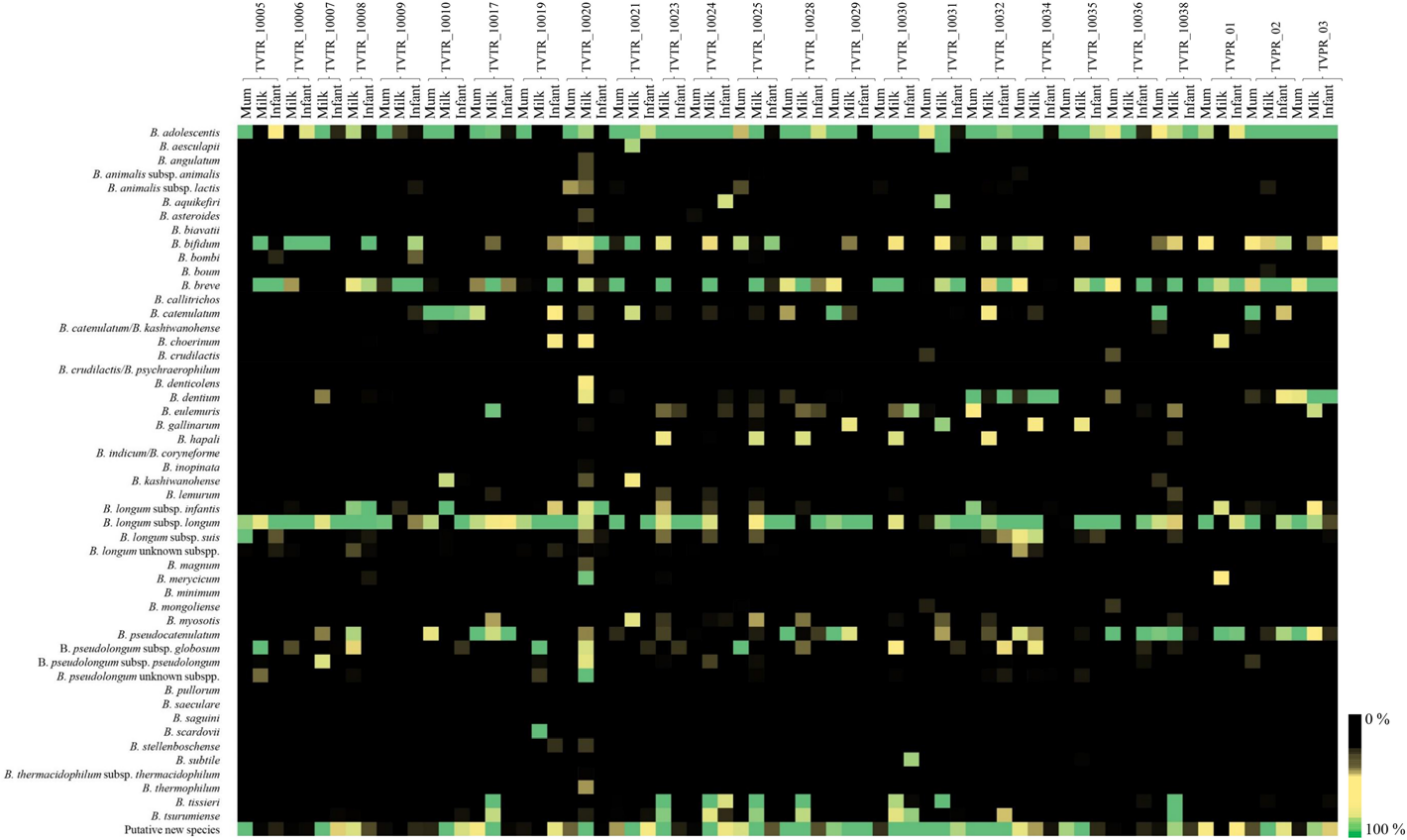
Profiling of the bifidobacterial communities of each CoupleID. The heat map represents the relative abundance of bifidobacterial species that were determined to be present in each sample. On the *left side*, all known and putative novel bifidobacterial species are reported. Sample origin and CoupleID codes are reported on the *top side* of the heat map

Bacterial transmission from mother to child has been proposed to occur during birth and as a consequence of breastfeeding [40]. In order to explore if a similar scenario is applicable to the bifidobacterial population, we compared bifidobacterial ITS profiles of infants with those of their corresponding mother’s fecal (obtained during or soon after delivery) and milk samples obtained 7 and/or 30 days after delivery (Fig. 2 and Additional file 2: Figure S1). Interestingly, the number of ITS-based OTUs shared by a given sample set ranged from three in TVPR_01 to 273 in TVTR_10032 (Additional file 1: Table S2). These ITS-based OTUs encompass members of the species *B. bifidum*, *B. adolescentis*, *B. dentium*, *B. breve*, *B. longum* spp., *B. pseudocatenulatum*, and *B. eulemuris* and OTUs displaying a level of similarity below 93% to any of the current known bifidobacterial (sub)species (Fig. 2 and Additional file 2: Figure S1). Furthermore, we highlighted that the most prevalent bifidobacterial species are *B. breve*, *B. longum* spp., *B. bifidum*, *B. adolescentis*, *B. dentium*, and *B. pseudocatenulatum*, all being identified in infant feces. These taxa were also found in the corresponding fecal and milk samples of the mother (Fig. 2 and Additional file 2: Figure S1). Notably, comparison of the ITS-based OTUs identified in the data sets indicated the presence of identical ITS-based OTUs in different sample pairs, implying that these identical sequences correspond to very closely related strains that are present in non-corresponding mother-infant dyads. We therefore decided to verify these findings using a cultivation approach where we isolated such strains followed by de novo genome sequencing of such bifidobacterial isolates.

### Isolation and genome sequencing of shared mother-child bifidobacterial strains

**Fig. 3:**
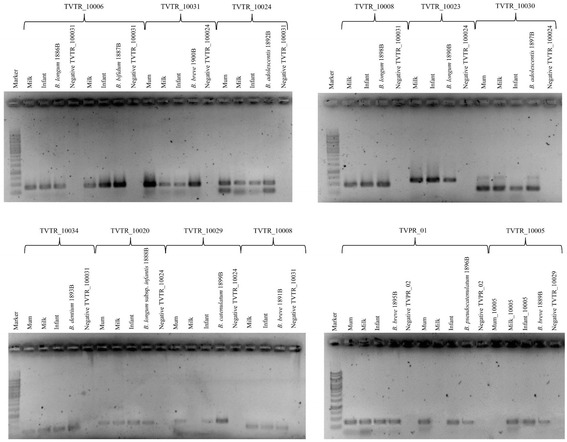
Evaluation of persistence of putative vertically transmitted strains in fecal samples of the infants and mothers as well as in milk samples by PCR assays. Marker lane is the Thermo Scientific GeneRuler 1 kb DNA Ladder. The reference strains used as positive controls for each CoupleID respectively are *B. longum* 1886B, *B. bifidum* 1887B, *B. breve* 1900B, *B. adolescentis* 1892B, *B. longum* 1898B, *B. longum* 1897B, *B. dentium* 1893B, *B. longum* subsp. *infantis* 1888B, *B. catenulatum* 1899B, *B. breve* 1891B, *B. breve* 1895B, *B. pseudocatenulatum* 1896B, and *B. breve* 1889B. Primer sequences are reported in Additional file 1: Table S3

In order to validate the notion that the identical bifidobacterial ITS-based OTUs that seem to be shared between mother-newborn pairs are the result of vertical transmission, we used 25 infant and mother stool samples, as well as milk samples, to isolate bifidobacteria employing a selective cultivation medium (Table 1). Isolated colonies were identified by PCR amplification of the 16S rRNA gene followed by amplicon sequencing as previously described by Turroni et al. [41]. This cultivation effort resulted in the isolation of 14 bifidobacterial strains, whose taxonomical identity perfectly matched those identified by bifidobacterial ITS profiling described above for the same samples (Fig. 2 and Additional file 2: Figure S1). These 14 isolated strains, representing different bifidobacterial species (Table 2), were then subjected to whole-genome sequencing using an Illumina-MiSeq platform. As outlined in Table 2, the predicted general features of these genomes appear to be similar to those generally observed for bifidobacteria [42]. Moreover, to identify the presence of these strains in stool samples of the corresponding mother, we performed a comparative genome analysis to identify the occurrence of specific, unique genes for each strain. Such analyses allowed the design of strain-specific primer pairs to be used in a PCR-based detection method involving DNA isolated from (mother’s) stool samples as a template (Additional file 1: Table S3). Notably, these experiments resulted in the detection of each of the infant stool-isolated and genome-sequenced strains in the fecal samples of the corresponding mother. In addition, we used isolated DNA from a mother stool sample of a non-corresponding CoupleID to detect false positive results (Fig. 3). Altogether, these data clearly corroborate the notion that these bifidobacterial isolates were obtained by infants via a maternal vertical transmission route (Fig. 3).

**Table 2:** General genome features of vertical transmitted bifidobacterial strains

	Couple ID	Biological origin	Contig	Genome length	% GC	Predicted ORFs	tRNA	rRNA
*Bifidobacterium longum* subsp. *longum* 1886B	TVTR_10006	Human milk	47	2473746 bp	61.14%	2137	62	3
*Bifidobacterium bifidum* 1887B	TVTR_10006	Human milk	21	2255543 bp	59.49%	1851	53	3
*Bifidobacterium longum* subsp. *infantis* 1888B	TVTR_10020	Infant stool	26	2579732 bp	59.40%	2212	55	3
*Bifidobacterium breve* 1889B	TVTR_10005	Infant stool	22	2344818 bp	59.66%	2037	53	2
*Bifidobacterium longum* subsp. *longum* 1890B	TVTR_10023	Infant stool	109	2341670 bp	59.55%	1955	60	2
*Bifidobacterium breve* 1891B	TVTR_10008	Infant stool	38	2089648 bp	59.70%	2115	54	2
*Bifidobacterium adolescentis* 1892B	TVTR_10024	Infant stool	11	2150850 bp	58.65%	1720	55	4
*Bifidobacterium dentium* 1893B	TVTR_10034	Infant stool	24	2271880 bp	58.74%	2070	56	4
*Bifidobacterium breve* 1895B	TVPR_01	Infant stool	17	20270860 bp	58.58%	1871	53	2
*Bifidobacterium pseudocatenulatum*1896B	TVPR_01	Human milk	15	2197471 bp	56.15%	1733	54	5
*Bifidobacterium longum* subsp. *longum* 1897B	TVTR_10030	Human milk	55	2453605 bp	59.50%	2143	55	2
*Bifidobacterium longum* subsp. *longum* 1898B	TVTR_10008	Infant stool	41	2474386 bp	59.47%	2049	57	3
*Bifidobacterium catenulatum* 1899B	TVTR_10029	Infant stool	14	2124599 bp	58.90%	1749	55	5
*Bifidobacterium breve* 1900B	TVTR_10031	Infant stool	22	2287865 bp	59.50%	1910	53	2

### Genetic adaptation of early bifidobacterial colonizers to the infant gut

Transmission of (bifido)bacteria from mother to her child may be possible through ingestion of mother’s milk [38, 39, 43, 44]. This human fluid represents a bacterial transmission medium that is ideal not only for microbiota dispersal but also for the provision of nutrients to the initial colonizers of the infant gut [10, 38, 39, 44, 45]. Human milk is a very rich source of glycans, including lactose and human milk oligosaccharides (HMOs) [46], which represent an important carbon and energy source for pioneering saccharolytic members of the infant gut microbiota, in particular bifidobacteria [47–49]. However, not all bifidobacteria are genetically adapted to utilize such milk-derived glycans [47, 50].

**Fig. 4:**
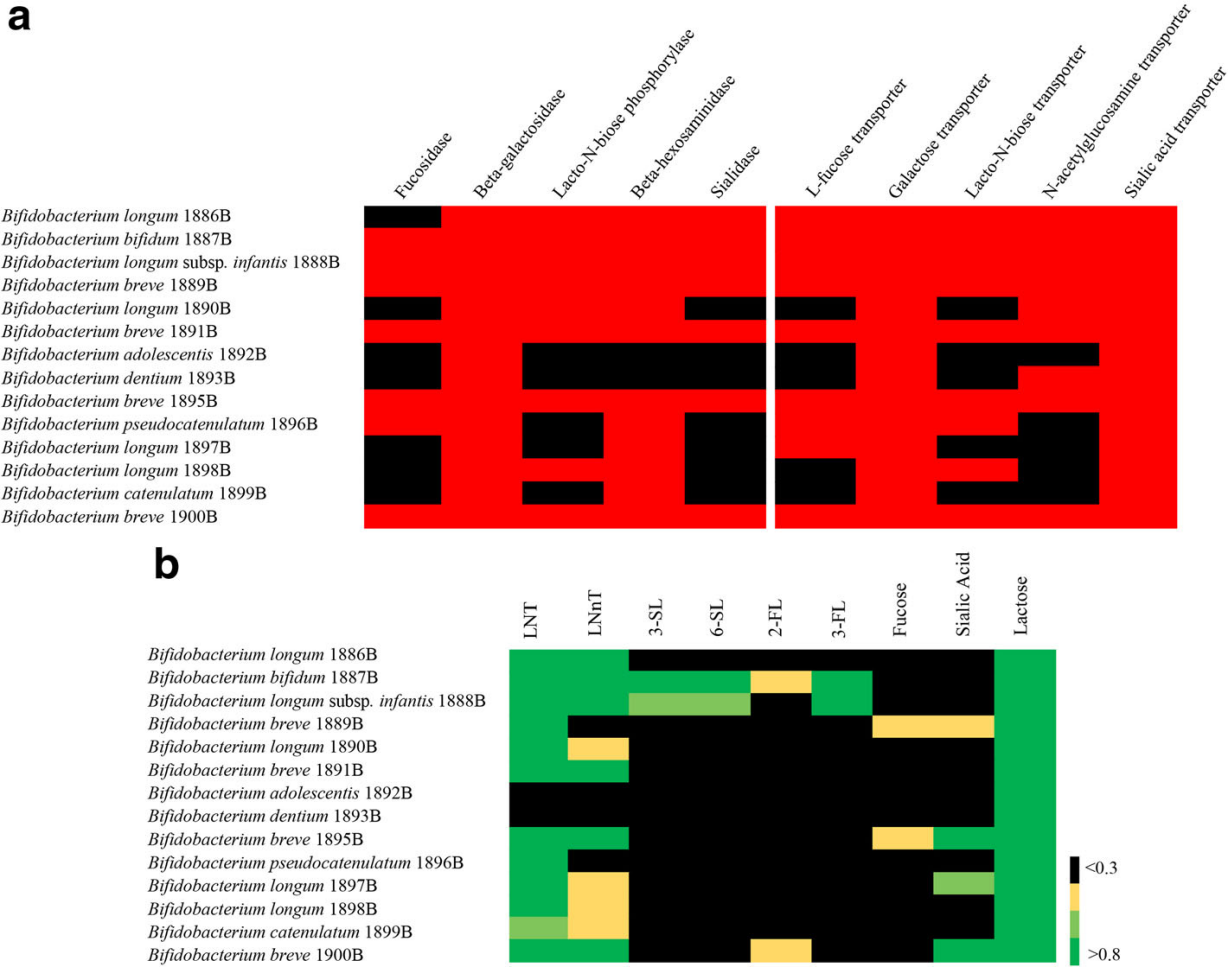
Human milk oligosaccharide degradation capabilities of vertically transmitted bifidobacterial strains. **a** Heat map representation of the presence/absence of genes encoding enzymes for degradation of milk oligosaccharides and transporters for uptake of the released compounds. *Red* and *black squares* represent the presence and absence of genes, respectively. **b** Growth profile on HMO-derived carbohydrates. *Black color* indicates that the final OD_600 nm_ is <0.3, *yellow* indicates the range of final OD_600 nm_ from 0.3 to 0.5, *light green* indicates the range between >0.5 and 0.8, and *green* indicates the final OD_600 nm_ >0.8

In order to identify the genomic repertoire involved in the utilization of HMO, lactose, and/or HMO-derived carbohydrates, such as fucose, lactose, sialic acid, fucosyllactose (2-FL and 3-FL), sialyllactose (3-SL and 6-SL), lacto-*N*-tetraose (LNT), and lacto-*N*-neotetraose (LNnT), we performed a comparative genomic analysis of all genomes decoded in this study. Our analysis revealed that six strains belonging to *B. longum*, *B. breve*, or *B. bifidum* possess a variety of genes allowing them to metabolize various HMO and HMO-derived glycans [47, 49–51] (Fig. 4a). In contrast, these genes are absent in the genomes of *B. adolescentis* 1892B and *B. dentium* 1893B, except for the genes coding for β-galactosidase (Fig. 4a). Moreover, this analysis revealed that putative fucosidase- and sialidase-encoding genes are absent in the examined *B. longum* genomes, except for that of *B. longum* 1886B, which encompasses an ORF (B1886_0565) specifying a predicted sialidase. Furthermore, no predicted sialidase-encoding genes were identified in the genome of *B. pseudocatenulatum* 1896B, while no homologs of the gene encoding lacto-*N*-biose phosphorylase were detected in the chromosomes of *B. pseudocatenulatum* 1896B, *B. longum* 1897B, or *B. catenulatum* 1899B.

In order to validate these in silico data, we performed growth experiments involving the bifidobacterial strains that we isolated in this study using an mMRS medium containing various HMOs and HMO-derived glycans, i.e., LNT, LNnT, 3-SL, 6-SL, 2-FL, 3-FL, fucose, sialic acid, and lactose, as the sole carbon source (Fig. 4b). Based on these culturing experiments, we observed that the *B. longum* strains, *B. bifidum* 1887B, and *B. breve* strains were able to grow (OD values greater than 0.3) on LNT, LNnT, and lactose, while *B. adolescentis* 1892B and *B. dentium* 1893B were unable to grow on these carbohydrates, except for lactose. Moreover, only *B. breve* 1889B and *B. breve* 1895B were able to utilize sialic acid and fucose, while *B. bifidum* 1887B and *B. longum* subsp. *infantis* 1888B were able to utilize 3-SL, 6-SL, and 3-FL. Finally, only *B. bifidum* 1887B was able to degrade 2-FL. Notably, these data are consistent with current knowledge on bifidobacterial HMO/HMO-derived glycan utilization abilities [47, 49, 51–55].

Interestingly, the results of these fermentation profiling experiments, correspond very well with those predicted from the in silico analyses of the genomes decoded in this study.

### Genome reconstruction of shared bifidobacteria based on in silico analyses of microbiomes

In order to verify the occurrence of identical bifidobacterial strains in the microbiota of an infant’s fecal sample and in that of the corresponding milk and/or fecal sample of the mother, a shotgun metagenomic analysis was performed of three infant samples (TVPR-01, TVPR-02, and TVPR-03) for which we observed a high number of potentially vertically transmitted bifidobacterial strains. Taxonomic assignments (based on publicly available bifidobacterial genomic data) of the obtained microbiome data (i.e., shotgun metagenomic reads) corresponding to the TVPR-01, TVPR-02, and TVPR-03 infant fecal samples showed that 2.09, 9.02, and 12.16% of these shotgun metagenomic reads were classified as bifidobacterial DNA, respectively (Additional file 1: Table S4). Furthermore, taxonomic classification of these data sets determined that in sample TVPR-01, 1.56% of reads was taxonomically assigned to *B. breve*, while for TVPR-02, 6.16% of reads were annotated as *B. dentium* sequences, and finally, in the case of TVPR-03, 8.21% of reads were shown to correspond to *B. dentium* (Additional file 1: Table S4). Moreover, we reconstructed the complete genome sequences of each of the abovementioned bifidobacterial strains, by an in silico analysis of these three shotgun metagenomics datasets using a previously described, in-house developed pipeline [11, 34].

Genomic comparison of the isolated *B. breve* 1895B and the reconstructed *B. breve* TVPR-01 clearly showed the isogenic nature of both genomes, exhibiting an ANI value of 99.99%. Thus, using three different approaches, i.e., ITS profiling, strain isolation from fecal samples, and genome reconstruction from shotgun metagenomic data, we were able to confirm the essentially identical genetic composition of these bifidobacterial strains. Furthermore, we verified, through PCR-amplification of unique gene (Additional file 1: Table S3), that *B. breve* 1895B/TVPR-01 was shared between mother and child (Fig. 3). Unfortunately, despite several attempts, we were not able to isolate the *B. dentium* TVPR-02 and *B. dentium* TVPR-03 strains, most likely because of exigent nutritional requirements of these strains. Nonetheless, PCR amplification attempts targeting specific genes of TVPR-02 and TVPR-03, which were identified by means of comparative genomic analyses of the TVPR-02 and TVPR-03 microbiome-reconstructed genomes against the nine publicly available *B. dentium* strains, and confirmed the presence of these strains in corresponding mothers and infants (Additional file 3: Figure S2 and Additional file 1: Table S3). In order to avoid false positive results, we used isolated DNA from a mother stool sample of a non-corresponding dataset (Additional file 3: Figure S2). Consequently, these data clearly support the assumption of a vertical maternal inheritance of the most abundant bifidobacterial strains identified in the gut microbiota of children, as previously described in other studies [11, 15].

### Bifidoprophages transmission from mother to child

**Fig. 5:**
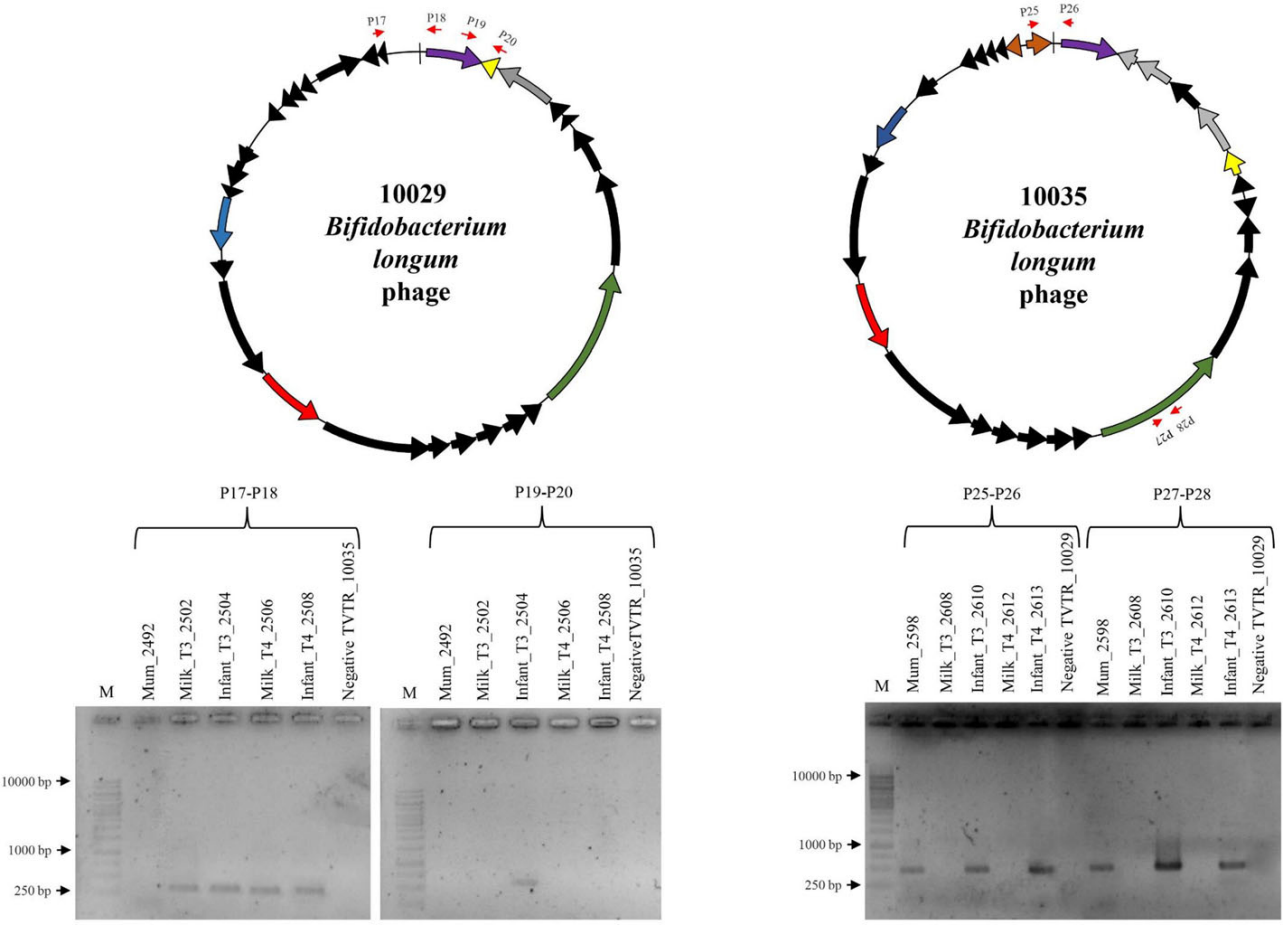
Vertically transmitted bifidophages from mothers to newborns. Indicated at the top of the figure are the genomic maps of the identified *B. longum* phage 10029 and 10035. The modular genomic structure is indicated by different patterns, which specifies their predicted function (*violet*, lysogeny module; *blue*, DNA replication; *red*, DNA packaging and head; *green*, tail and tail fiber; *yellow*, lysis module; *black arrows*, hypothetical protein; *grey arrows*, similar to bacterial protein). On the bottom are reported the presence of the phage within the mother’s and infant’s metagenomic samples through PCR analysis. Primer sequences are reported in Additional file 1: Table S5

Bacteriophages may also be transmitted from mother to child during the early phases of life, provided that the corresponding hosts for such phages are also passed across. Thus, we searched shotgun metagenomics data, obtained from stool samples of various mother and corresponding child and milk sample sets, for sequences that matched with known bacteriophages infecting bifidobacteria, i.e., bifidophages [24], focusing on datasets in which we had already identified bifidobacterial transmission. In order to identify the presence of both bifidophages as well as bifidoprophages, we scanned all obtained metagenomic datasets using a previously developed bioinformatics pipeline [11, 34]. Putative bifidobacterial prophage sequences were identified by manual examination of all genes belonging to the contigs attributed to bifidobacterial (sub)species [24, 56, 57]. The bifidobacterial phage/prophage screening revealed the presence of 21 putative bifido(pro)phages within nine samples that exhibit integrated or circular phage genome sequences (Table 3). A manually curated screening of the reconstructed phages unveiled the circular status of their genomes for eight bifidophages, highlighting overlapping sequence ends within the assembled contigs (Additional file 4: Figure S3). Interestingly, taxonomical gene classification based on NCBI RefSeq database matches reveal that these phages were predicted to belong to several bifidobacterial (sub)species, i.e., *B. adolescentis*, *B. breve*, *B. dentium*, *B. longum*, and *B. pseudocatenulatum* (Additional file 4: Figure S3). Since it was not possible to retrieve sequences of these identified bifidophages from each of the three components of a given metagenomic dataset (i.e., a corresponding mother and infant fecal samples and associated milk sample), we decided to utilize a PCR-based approach involving a PCR primer pair that was designed on unique sequences of a given phage (Additional file 1: Table S5). Furthermore, a second PCR primer pair was designed on the contig edges, in order to determine if the phage DNA was circular/concatameric or integrated into the host chromosome (Additional file 1: Table S5). As shown in Fig. 5, *B. longum* phage 10029 was identified in the fecal samples T3 and T4 of the same child as well as in the milk samples of the corresponding mother. While the use of the primer pair (P19-P20) results in the amplification of phage DNA in infant and milk samples, the second primer pair (P17-P18) unveiled the presence of a circular phage, yet only in sample T3 of the child. These data clearly indicates that this bifido(pro)phage was vertically transmitted by the mother through breastfeeding as part of the bifidobacterial host and was then apparently induced in the gut of the newborn. Furthermore, for samples Mum_2598, Infant_T3_2610, and Infant_T4_2613, the PCR amplification of both primer sets of the *B. longum* phage 10035 produced an amplicon (Fig. 5). This finding suggests the presence of the same bifidophage in both mother and infant microbiome, expanding the previous view of bifidobacterial phages multiplying in the infant gut thereby perhaps limiting overgrowth by their bifidobacterial host [24].

**Table 3:** Identified bifidophage/bifidoprophages within metagenomes

Samples	Species assignment	Genome status
TVPR3B	*B. adolescentis*	Integrated
	*B. adolescentis*	Integrated
	*B. adolescentis*	Circular
C10009IS2149FEt5	*B. bifidum*	Integrated
	*B. bifidum*	Unknown
	*B. breve*	Integrated
	*B. breve*	Integrated
C10009MS2134FEt0	*B. adolescentis*	Circular
C10019IS2329FEt5	*B. breve*	Circular
C10029IS2503FEt3	*B. longum* subsp. *longum*	Unknown
	*B. longum* subsp. *longum*	Unknown
	*B. longum* subsp. *longum*	Unknown
	*B. longum* subsp. *longum*	Unknown
	*B. longum* subsp. *longum*	Circular
	n.d.	Circular
C10029MS2492FEt0	*B. pseudolongum*	Circular
C10035IS2609FEt3	*B. longum* subsp. *longum*	Integrated
	*B. longum* subsp. *longum* ^a^	Circular
C10035IS2913FEt4	*B. breve*	Circular
	*B. longum* subsp. *longum* ^a^	Circular
C10035MS2598FEt0	*B. longum* subsp. *longum*	Unknown

*n.d.* Bifidobacterial species not detected, *Unknown* genome-status unknown

^a^ *B. longum* phages that share the same genomic sequences between samples

## Conclusions

Passage through the birth canal during delivery and subsequent breastfeeding are considered important events that affect microbial colonization of the gastrointestinal tract of the newborn [15, 45, 58, 59]. In this study, an NGS approach was employed to confirm the existence of a vertical transmission route of bifidobacterial communities in 25 mother and newborn pairs. Furthermore, our analyses highlight how bifidobacteria, being one of the dominant members of the infant gut microbiota, are inherited from the mother through a vertical transmission route, and imply human milk as a potential vehicle to facilitate this acquisition. Milk-mediated transmission of certain bifidobacterial strains may be supported by their ability to utilize HMOs and/or HMO-derived glycans. It is plausible that the first microbial colonizers of the human gut include commensals that are able to metabolize such milk glycans, such as the bifidobacterial strains belonging to *B. bifidum* species. However, these initial bifidobacterial colonizers may be important to promote the establishment of bifidobacterial colonizers that are not by themselves able to degrade and thus utilize milk carbohydrates. These bifidobacterial colonizers can access HMO degradation products, such as sialic acid and fucose, employing a cross-feeding behavior, which has been recently described [54, 55, 60, 61].

Another crucial microbial player involved in the establishment and maintenance of a so-called climax gut microbiota encompasses bacteriophages [19, 62]. Very recently, bifidophages have been discovered and their phage particles have been morphologically characterized [24]. However, their inheritance in the infant gut from the gut virome of the respective mother was only hypothesized but never experimentally demonstrated. Our data clearly highlight the existence of a vertical transfer route for bifidophages, thereby facilitating their spread from mother to newborn.

## Additional files

Additional file 1: **Table S1.** Taxonomy and filtering report of samples included in this study. **Table S2** ITS-based OTUs shared by an entire sample sets. **Table S3** List of bifidobacterial strain-specific primers. **Table S4** Metagenomic reads classification of samples TVPR-01, TVPR-02, and TVPR-03. **Table S5** List of specific primers for each bifidophage/bifidoprophages identified. (DOCX 37 kb)

Additional file 2: **Figure S1.** ITS-based OTUs shared between mother-milk-infant sample sets. The bar plot represents the percentage of the total bifidobacterial population found in mother-milk, mother-infant, milk-infant and mother-milk-infant samples. (JPG 1306 kb)

Additional file 3: **Figure S2.** Evaluation of presence of TVPR-02 and TVPR-03 microbiome-reconstructed genomes in fecal samples of the infants and mothers as well as in milk samples. Primer sequences are reported in Additional file 1: Table S2. (JPG 235 kb)

Additional file 4: **Figure S3.** Identified bifidophage/bifidoprophage within the mother’s and infant’s metagenomic samples. Genomic maps of phages recall their modular genomic structure indicated by different patterns, which specifies their predicted function (*violet*: lysogeny module; *blue*: DNA replication; *red*: DNA packaging and head; *green*: tail and tail fiber; *yellow*: lysis module; *black arrows*: hypothetical protein; *grey arrows*: similar to bacterial protein). For each phage is reported the presence within the mother’s and infant’s metagenomic samples through PCR analysis. Primer sequences are reported in Additional file 1: Table S5. (JPG 1173 kb)

## Abbreviations

2-FL: 2-Fucosyllactose
3-FL: 3-Fucosyllactose
3-SL: 3-Sialyllactose
6-SL: 6-Sialyllactose
GIT: Gastrointestinal tract
HMO: Human milk oligosaccharides
ITS: Internal transcribed spacer
LNnT: Lacto-*N*-neotetraose
LNT: Lacto-*N*-tetraose
MRS: de Man-Rogosa-Sharpe medium
OTUs: Operational taxonomic units
PBS: Phosphate-buffered saline
qRT-PCR: Quantitative real-time PCR
