# Supplementary material for: Maternal inheritance of bifidobacterial communities and bifidophages in infants through vertical transmission
Source: Microbiome. 2017 Jun 26;5:66. doi: 10.1186/s40168-017-0282-6 (PMC5485682; doi:10.1186/s40168-017-0282-6)
Supplement: Supplementary file 1 — Table S1. Taxonomy and filtering report of samples included in this study. Table S2 ITS-based OTUs shared by an entire sample sets. Table S3 List of bifidobacterial strain-specific primers. Table S4 Metagenomic reads classification of samples TVPR-01, TVPR-02, and TVPR-03. Table S5 List of specific primers for each bifidophage/bifidoprophages identified. (DOCX 37 kb) [file 40168_2017_282_MOESM1_ESM.docx]

**Table S1.** Taxonomy and filtering report of samples included in this study.

| **Samples** | | | **Number of merged pe reads** | **Human sequences** | **Homopolymers > 7** | **Mismatch in primers >1** | **Reverse primer not found** | **Final Read Number** |
| --- | --- | --- | --- | --- | --- | --- | --- | --- |
| **TVTR_10005** | **Mum-T0** | **2062** | 102396 | 0 | 5 | 1629 | 767 | 99995 |
| **TVTR_10005** | **Milk-T3** | **2072** | 13049 | 1 | 6 | 380 | 153 | 12539 |
| **TVTR_10005** | **Infant-T3** | **2074** | 25693 | 0 | 0 | 414 | 154 | 25071 |
| **TVTR_10005** | **Milk-T4** | **2076** | 6510 | 0 | 1 | 165 | 39 | 6305 |
| **TVTR_10005** | **Infant-T4** | **2078** | 33322 | 0 | 0 | 783 | 218 | 32321 |
| **TVTR_10006** | **Milk-T3** | **2090** | 68420 | 0 | 38 | 1774 | 537 | 66071 |
| **TVTR_10006** | **Infant-T3** | **2092** | 44510 | 0 | 0 | 1158 | 369 | 42956 |
| **TVTR_10006** | **Milk-T4** | **2094** | 9100 | 3 | 0 | 307 | 44 | 8746 |
| **TVTR_10006** | **Infant-T4** | **2096** | 97443 | 0 | 15 | 2858 | 711 | 93859 |
| **TVTR_10007** | **Milk-T3** | **2108** | 79029 | 1 | 9 | 1131 | 592 | 77296 |
| **TVTR_10007** | **Infant-T3** | **2110** | 117282 | 0 | 3 | 1913 | 1073 | 114282 |
| **TVTR_10007** | **Milk-T4** | **2112** | 204852 | 3 | 36 | 2903 | 1571 | 200339 |
| **TVTR_10007** | **Infant-T4** | **2114** | 102806 | 0 | 1 | 1616 | 894 | 100294 |
| **TVTR_10008** | **Milk-T3** | **2126** | 98614 | 1 | 18 | 2455 | 727 | 95413 |
| **TVTR_10008** | **Infant-T3** | **2128** | 26375 | 0 | 0 | 961 | 246 | 25168 |
| **TVTR_10008** | **Milk-T4** | **2130** | 42276 | 0 | 2 | 1490 | 221 | 40563 |
| **TVTR_10008** | **Infant-T4** | **2132** | 74380 | 0 | 12 | 2744 | 698 | 70926 |
| **TVTR_10009** | **Mum-T0** | **2134** | 44299 | 0 | 2 | 794 | 325 | 43178 |
| **TVTR_10009** | **Milk-T3** | **2144** | 3707 | 3 | 0 | 96 | 30 | 3578 |
| **TVTR_10009** | **Infant-T3** | **2146** | 76312 | 0 | 3 | 1999 | 603 | 73707 |
| **TVTR_10009** | **Milk-T4** | **2148** | 51540 | 0 | 0 | 956 | 311 | 50273 |
| **TVTR_10009** | **Infant-T4** | **2150** | 20687 | 1 | 0 | 429 | 133 | 20124 |
| **TVTR_10010** | **Mum-T0** | **2152** | 91691 | 0 | 2 | 1717 | 817 | 89155 |
| **TVTR_10010** | **Milk-T3** | **2162** | 72562 | 2 | 1 | 1187 | 521 | 70851 |
| **TVTR_10010** | **Infant-T3** | **2164** | 87379 | 0 | 5 | 2107 | 683 | 84584 |
| **TVTR_10010** | **Milk-T4** | **2166** | 83604 | 0 | 31 | 1266 | 637 | 81670 |
| **TVTR_10010** | **Infant-T4** | **2168** | 93979 | 57 | 13 | 1477 | 789 | 91643 |
| **TVTR_10017** | **Mum-T0** | **2278** | 89586 | 1 | 0 | 1284 | 704 | 71894 |
| **TVTR_10017** | **Milk-T3** | **2287** | 58113 | 0 | 3 | 1025 | 426 | 56659 |
| **TVTR_10017** | **Infant-T3** | **2290** | 60015 | 0 | 1 | 1015 | 464 | 58535 |
| **TVTR_10017** | **Milk-T4** | **2278** | 73886 | 1 | 2 | 1284 | 704 | 71894 |
| **TVTR_10017** | **Infant-T4** | **2292** | 70404 | 0 | 11 | 1090 | 518 | 68785 |
| **TVTR_10017** | **Infant** | **2293** | 60429 | 0 | 1 | 980 | 659 | 58789 |
| **TVTR_10019** | **Mum-T0** | **2314** | 115302 | 0 | 0 | 1895 | 918 | 112489 |
| **TVTR_10019** | **Milk-T3** | **2324** | 72273 | 3 | 122 | 1047 | 507 | 70594 |
| **TVTR_10019** | **Infant-T3** | **2326** | 53137 | 0 | 7 | 1389 | 394 | 51347 |
| **TVTR_10019** | **Milk-T4** | **2328** | 81959 | 2 | 49 | 2157 | 600 | 79151 |
| **TVTR_10019** | **Infant-T4** | **2330** | 34494 | 4 | 1 | 731 | 205 | 33553 |
| **TVTR_10020** | **Mum-T0** | **2332** | 41438 | 0 | 0 | 675 | 228 | 40535 |
| **TVTR_10020** | **Milk-T3** | **2342** | 15259 | 0 | 2 | 286 | 119 | 14852 |
| **TVTR_10020** | **Infant-T3** | **2344** | 115194 | 0 | 27 | 1418 | 618 | 113130 |
| **TVTR_10020** | **Milk-T4** | **2346** | 8077 | 0 | 12 | 438 | 79 | 7548 |
| **TVTR_10020** | **Infant-T4** | **2347** | 115194 | 0 | 27 | 1418 | 618 | 113130 |
| **TVTR_10021** | **Mum-T0** | **2350** | 34319 | 0 | 4 | 678 | 345 | 33292 |
| **TVTR_10021** | **Milk-T3** | **2360** | 99020 | 0 | 2 | 2716 | 729 | 95573 |
| **TVTR_10021** | **Infant-T3** | **2362** | 65248 | 2 | 4 | 1780 | 567 | 62895 |
| **TVTR_10021** | **Milk-T4** | **2364** | 78417 | 3 | 13 | 1258 | 700 | 76443 |
| **TVTR_10021** | **Infant-T4** | **2365** | 78716 | 0 | 5 | 1371 | 676 | 76664 |
| **TVTR_10023** | **Milk-T3** | **2396** | 69176 | 0 | 2 | 1242 | 531 | 67401 |
| **TVTR_10023** | **Infant-T3** | **2398** | 56630 | 0 | 1 | 1071 | 449 | 55109 |
| **TVTR_10023** | **Milk-T4** | **2400** | 89433 | 0 | 4 | 1607 | 751 | 87071 |
| **TVTR_10023** | **Infant-T4** | **2401** | 62860 | 1 | 0 | 876 | 417 | 61566 |
| **TVTR_10024** | **Mum-T0** | **2404** | 48966 | 0 | 7 | 1007 | 330 | 47622 |
| **TVTR_10024** | **Milk-T3** | **2414** | 106617 | 4 | 9 | 1691 | 766 | 104148 |
| **TVTR_10024** | **Infant-T3** | **2416** | 109560 | 0 | 3 | 1615 | 772 | 107170 |
| **TVTR_10024** | **Milk-T4** | **2418** | 54893 | 0 | 1 | 882 | 410 | 53600 |
| **TVTR_10024** | **Infant-T4** | **2419** | 87058 | 2 | 3 | 1536 | 606 | 84911 |
| **TVTR_10025** | **Mum-T0** | **2422** | 82704 | 0 | 0 | 2168 | 667 | 79869 |
| **TVTR_10025** | **Milk-T3** | **2432** | 104690 | 2 | 3 | 1751 | 772 | 102162 |
| **TVTR_10025** | **Infant-T3** | **2434** | 78564 | 0 | 1 | 1375 | 697 | 76491 |
| **TVTR_10025** | **Milk-T4** | **2436** | 89134 | 1 | 4 | 1450 | 702 | 86977 |
| **TVTR_10025** | **Infant-T4** | **2437** | 78036 | 0 | 3 | 1351 | 716 | 75966 |
| **TVTR_10028** | **Mum-T0** | **2476** | 96221 | 0 | 3 | 1331 | 580 | 94307 |
| **TVTR_10028** | **Milk-T3** | **2486** | 74473 | 1 | 5 | 1192 | 597 | 72678 |
| **TVTR_10028** | **Infant-T3** | **2487** | 78342 | 1 | 3 | 1236 | 651 | 76451 |
| **TVTR_10028** | **Milk-T4** | **2490** | 65970 | 0 | 4 | 1190 | 540 | 64236 |
| **TVTR_10028** | **Infant-T4** | **2491** | 57952 | 0 | 0 | 770 | 349 | 56833 |
| **TVTR_10029** | **Mum-T0** | **2492** | 82353 | 0 | 14 | 1565 | 883 | 79891 |
| **TVTR_10029** | **Milk-T3** | **2502** | 87953 | 1 | 5 | 1485 | 640 | 85822 |
| **TVTR_10029** | **Infant-T3** | **2504** | 98672 | 1 | 2 | 1589 | 951 | 96129 |
| **TVTR_10029** | **Milk-T4** | **2506** | 84255 | 5 | 30 | 52941 | 242 | 31037 |
| **TVTR_10029** | **Infant-T4** | **2508** | 33751 | 0 | 0 | 619 | 243 | 32889 |
| **TVTR_10030** | **Mum-T0** | **2509** | 54162 | 0 | 2 | 984 | 373 | 52803 |
| **TVTR_10030** | **Milk-T3** | **2519** | 37585 | 4 | 0 | 584 | 262 | 36735 |
| **TVTR_10030** | **Infant-T3** | **2521** | 123953 | 2 | 17 | 1796 | 1026 | 121112 |
| **TVTR_10030** | **Milk-T4** | **2523** | 65102 | 0 | 2 | 1109 | 531 | 63460 |
| **TVTR_10030** | **Infant-T4** | **2525** | 77173 | 0 | 1 | 1293 | 600 | 75279 |
| **TVTR_10031** | **Mum-T0** | **2526** | 123106 | 0 | 233 | 2200 | 1138 | 119535 |
| **TVTR_10031** | **Milk-T3** | **2536** | 123606 | 0 | 12 | 1740 | 874 | 120980 |
| **TVTR_10031** | **Infant-T3** | **2538** | 3473 | 0 | 0 | 66 | 1370 | 332 |
| **TVTR_10031** | **Milk-T4** | **2540** | 139409 | 3 | 28 | 2076 | 1154 | 136148 |
| **TVTR_10031** | **Infant-T4** | **2541** | 87068 | 0 | 7 | 1080 | 500 | 85481 |
| **TVTR_10032** | **Mum-T0** | **2544** | 104083 | 1 | 13 | 1651 | 842 | 101576 |
| **TVTR_10032** | **Milk-T3** | **2554** | 96766 | 3 | 3 | 1314 | 637 | 94809 |
| **TVTR_10032** | **Infant-T3** | **2556** | 83442 | 4 | 4 | 1137 | 513 | 81784 |
| **TVTR_10032** | **Milk-T4** | **2558** | 41767 | 1 | 11 | 839 | 358 | 40558 |
| **TVTR_10032** | **Infant-T4** | **2559** | 90313 | 0 | 16 | 1577 | 814 | 87904 |
| **TVTR_10034** | **Mum-T0** | **2580** | 73512 | 0 | 4 | 1162 | 713 | 71633 |
| **TVTR_10034** | **Milk-T3** | **2590** | 120774 | 0 | 2 | 1770 | 914 | 118088 |
| **TVTR_10034** | **Infant-T3** | **2592** | 74245 | 1 | 0 | 1018 | 516 | 72710 |
| **TVTR_10034** | **Milk-T4** | **2594** | 42785 | 0 | 1 | 623 | 292 | 41869 |
| **TVTR_10034** | **Infant-T4** | **2595** | 79359 | 0 | 0 | 1286 | 684 | 77389 |
| **TVTR_10035** | **Mum-T0** | **2598** | 43379 | 0 | 3 | 921 | 315 | 42140 |
| **TVTR_10035** | **Milk-T3** | **2608** | 30586 | 0 | 7 | 551 | 266 | 29762 |
| **TVTR_10035** | **Infant-T3** | **2610** | 99103 | 1 | 4 | 1572 | 817 | 96709 |
| **TVTR_10035** | **Milk-T4** | **2612** | 100218 | 1 | 8 | 1497 | 737 | 97975 |
| **TVTR_10035** | **Infant-T4** | **2613** | 116329 | 0 | 4 | 1919 | 992 | 113414 |
| **TVTR_10036** | **Mum-T0** | **2616** | 91545 | 0 | 4 | 1346 | 761 | 89434 |
| **TVTR_10036** | **Milk-T3** | **2626** | 32089 | 1 | 3 | 506 | 239 | 31340 |
| **TVTR_10036** | **Infant-T3** | **2628** | 97746 | 3 | 3 | 1530 | 778 | 95432 |
| **TVTR_10036** | **Milk-T4** | **2630** | 54151 | 0 | 0 | 895 | 431 | 52825 |
| **TVTR_10036** | **Infant-T4** | **2632** | 79671 | 2 | 0 | 1189 | 502 | 77978 |
| **TVTR_10038** | **Mum-T0** | **2652** | 77794 | 0 | 0 | 1745 | 775 | 75274 |
| **TVTR_10038** | **Milk-T3** | **2662** | 74545 | 0 | 01 | 1172 | 537 | 72835 |
| **TVTR_10038** | **Infant-T3** | **2664** | 62963 | 0 | 0 | 1027 | 478 | 61458 |
| **TVTR_10038** | **Milk-T4** | **2666** | 54393 | 0 | 0 | 859 | 403 | 53131 |
| **TVTR_10038** | **Infant-T4** | **2668** | 77244 | 0 | 4 | 1142 | 544 | 75554 |
| **TVPR_01** | **Mum-T0** | **3000** | 32691 | 0 | 0 | 30489 | 2 | 2200 |
| **TVPR_01** | **Milk-T4** | **3001** | 121789 | 0 | 1 | 120687 | 0 | 1102 |
| **TVPR_01** | **Infant-T4** | **3002** | 116790 | 0 | 0 | 115566 | 0 | 1224 |
| **TVPR_02** | **Mum-T0** | **3006** | 84008 | 2 | 2 | 3595 | 62 | 80347 |
| **TVPR_02** | **Milk-T3** | **3007** | 51210 | 0 | 4 | 35650 | 109 | 15447 |
| **TVPR_02** | **Infant-T3** | **3008** | 59191 | 0 | 13 | 16131 | 239 | 36427 |
| **TVPR_02** | **Milk-T4** | **3009** | 49420 | 6 | 1 | 39756 | 75 | 9580 |
| **TVPR_02** | **Infant-T4** | **3010** | 96942 | 75 | 6 | 2382 | 77 | 94402 |
| **TVPR_03** | **Mum-T0** | **3011** | 59980 | 26 | 0 | 52377 | 94 | 7479 |
| **TVPR_03** | **Milk-T4** | **3012** | 51210 | 0 | 4 | 35650 | 109 | 15447 |
| **TVPR_03** | **Infant-T4** | **3013** | 47375 | 2 | 3 | 35803 | 18 | 11549 |

**Table S2.** ITS-based OTUs shared by an entire sample sets.

|  | Mum-Milk | Mum-Infant | Milk-Infant | Mum-Milk-Infant |
| --- | --- | --- | --- | --- |
| TVTR_10005 | 139 | 14 | 160 | 13 |
| TVTR_10006 | n.d. | n.d. | 222 | n.d. |
| TVTR_10007 | n.d. | n.d. | 161 | n.d. |
| TVTR_10008 | n.d. | n.d. | 295 | n.d. |
| TVTR_10009 | 12 | 20 | 200 | 9 |
| TVTR_10010 | 21 | 6 | 348 | 4 |
| TVTR_10017 | 245 | 516 | 202 | 161 |
| TVTR_10019 | 123 | 109 | 233 | 96 |
| TVTR_10020 | 150 | 58 | 54 | 13 |
| TVTR_10021 | 187 | 404 | 127 | 105 |
| TVTR_10023 | n.d. | n.d. | 174 | n.d. |
| TVTR_10024 | 78 | 400 | 230 | 12 |
| TVTR_10025 | 73 | 447 | 51 | 29 |
| TVTR_10028 | 149 | 301 | 32 | 15 |
| TVTR_10029 | 314 | 360 | 222 | 193 |
| TVTR_10030 | 115 | 22 | 588 | 15 |
| TVTR_10031 | 368 | 135 | 474 | 91 |
| TVTR_10032 | 368 | 616 | 531 | 273 |
| TVTR_10034 | 330 | 18 | 189 | 11 |
| TVTR_10035 | 229 | 55 | 503 | 49 |
| TVTR_10036 | 169 | 398 | 185 | 81 |
| TVTR_10038 | 152 | 17 | 101 | 15 |
| TVPR_01 | 3 | 5 | 337 | 3 |
| TVPR_02 | 11 | 5 | 16 | 4 |
| TVPR_03 | 199 | 32 | 151 | 32 |

n.d. = not determined

**Table S3.** List of bifidobacterial strain-specific primers.

| Primer | Sequence |
| --- | --- |
| B1892_0715-0717 Fw | AACAAGACGCCGAAGATGAC |
| B1892_0715-0717 Rev | GCTGGCAGAAGATCAAGGAG |
| B1887_0177 Fw | ACTTGTTCCGAATCCACAGC |
| B1887_0177 Rev | GGCAGGCTAACCATAACAGC |
| B1889_0236-0237 Fw | CTGATCCACCATGTGACGAC |
| B1889_0236-0237 Rev | ACAACCTCAGCCATTGGAAC |
| B1891_0745 Fw | TGAGCACGCGACGTAATATC |
| B1891_0745 Rev | CTGATGGCGACCATACTGTG |
| BBR7E_0534 Fw | AGCGACGATATGATGCAATG |
| BBR7E_0534 Rev | CGTGAATACGCTGCACAGTC |
| B1900_1881 Fw | TGCTCGGCATATTCATCATC |
| B1900_1881 Rev | ATAGCGTCATCAAGGACACG |
| B1899_0751 Fw | AGCTGAACAATCGGATCTGC |
| B1899_0751 Rev | GCAGGTAGTTCAGGCTCGAC |
| B1893_0736 Fw | GCAGGCCTCTCAAGAATTAGC |
| B1893_0736 Rev | GGAGGCCTTGTTGAGTTCAG |
| B1886_0443 Fw | AAGCCAAGGACATGTTCGAC |
| B1886_0443 Rev | TGGTGTATCTGGCGTTCTTG |
| B1890_0996 Fw | ACAAGAGGAAGATCGCGAAG |
| B1890_0996 Rev | GGTGCTAGTCATGACCGTTG |
| B1897_1308 Fw | CCGGCTATATCGTGATGCTC |
| B1897_1308 Rev | TCGCCAAGTCTCCAATTAAC |
| B1898_0685 Fw | GACGCGCAAGGTTCAATAAC |
| B1898_0685 Rev | ACTATACAATGCGCCGTTGG |
| B1888_0673 Fw | GCCTCGCGAAGAATATGAAG |
| B1888_0673 Rev | TGACCAGTAGCGGACGTAGC |
| B1896_0729 Fw | GTCCGACGGAATGTTACGC |
| B1896_0729 Rev | GGAATCCTTGAGTCGCTCTC |
| *B.dentium*TVPR-02 Fw | ATGGACGTCTGGTTCAGGTC |
| *B.dentium*TVPR-02 Rev | ATGCTGCCGTTGTAGTCCTC |
| *B.dentium*TVPR-03 Fw | GCCTCTCTGAAGGATCAACG |
| *B.dentium*TVPR-03 Rev | GCCTGGAGCGACTTCAATAG |

| **Table S4.** Metagenomic reads classification of samples TVPR-01, TVPR-02 and TVPR-03 | | | | | | |
| --- | --- | --- | --- | --- | --- | --- |
| **Genera** | **TVPR-01** | | **TVPR-02** | | **TVPR-03** | |
|  | *Veillonella* | 43.9% | *Escherichia* | 42.4% | *Veillonella* | 30.7% |
|  | *Haemophilus* | 11.1% | *Shigella* | 25.5% | *Clostridium* | 20.5% |
|  | *Enterobacter* | 9.0% | *Bifidobacterium* | 9.0% | *Bifidobacterium* | 12.2% |
|  | *Bacteroides* | 3.2% | *Cutibacterium* | 1.6% | *Escherichia* | 9.4% |
|  | *Streptococcus* | 2.5% | *Myoviridae* | 1.3% | *Shigella* | 5.6% |
|  | *Bifidobacterium* | 2.1% | *Siphoviridae* | 1.2% | *Lachnospiraceae* | 1.5% |
|  | *Klebsiella* | 1.8% | *Actinomyces* | 1.1% | *Candidatus* | 1.2% |
|  | *Clostridium* | 1.5% | *Enterobacteriaceae* | 1.1% | *Methanobrevibacter* | 1.0% |
|  | *Candidatus* | 1.2% |  |  |  |  |
|  | *Clostridioides* | 1.2% |  |  |  |  |
|  | *Methanobrevibacter* | 1.0% |  |  |  |  |
|  |  |  |  |  |  |  |
| **Species** | **TVPR-01** | | **TVPR-02** | | **TVPR-03** | |
|  | *Veillonella seminalis* | 33.5% | *Shigella dysenteriae* Sd197 | 22.9% | *Veillonella seminalis* | 29.8% |
|  | *Enterobacter aerogenes* KCTC 2190 | 6.9% | *Escherichia coli* | 16.5% | *Clostridium paraputrificum* | 17.5% |
|  | *Veillonella parvula* | 4.7% | *Escherichia coli* str. K-12 substr. MG1655 | 13.5% | *Bifidobacterium dentium* | 8.2% |
|  | *Haemophilus sp.* | 4.2% | *Bifidobacterium dentium* | 6.2% | *Shigella dysenteriae* Sd197 | 5.0% |
|  | *Haemophilus parainfluenzae* | 4.0% | *Escherichia coli* UMN026 | 5.0% | *Escherichia coli* | 3.1% |
|  | *Veillonella dispar* | 1.8% | *Escherichia coli* O83:H1 str. NRG 857C | 2.9% | *Escherichia coli str.* K-12 substr. MG1655 | 2.8% |
|  | *Enterobacter aerogenes* | 1.7% | *Shigella flexneri* 2a str. 301 | 1.8% | *Escherichia coli* UMN026 | 2.0% |
|  | *Bifidobacterium breve* | 1.6% | *Cutibacterium avidum* | 1.6% | *Bifidobacterium* sp. | 1.6% |
|  | *Clostridioides difficile* 630 | 1.0% | *Escherichia coli* O157:H7 str. Sakai | 1.3% | *Lachnospiraceae bacterium* mt14 | 1.4% |
|  | *Veillonella sp.* | 1.0% | *Bifidobacterium* sp. | 1.2% | *Clostridium* sp. | 1.2% |
|  |  |  | *Escherichia coli* O104:H4 str. 2011C-3493 | 1.1% | *Clostridium botulinum* | 1.0% |
|  |  |  | *Escherichia coli* IAI39 | 1.1% |  |  |
|  |  |  | *Enterobacteriaceae* sp. | 1.0% |  |  |
| Only percentage above 1% are showed. | |  |  |  |  |  |

**Table S5.** List of specific primers for each bifidophage/bifidoprophages identified.

| Primer | Name | Sequence |
| --- | --- | --- |
| P1 | TVPR3PhageHolin_Fw | AGCGAGATAGCGAAGGTGTC |
| P2 | TVPR3PhageHolin_Rev | TGGAAGGACAACGACATCAC |
| P3 | TVPR3PhageTail_Fw | CCTACACGCAGATCGTCATC |
| P4 | TVPR3PhageTail_Rev | GTGCAGCATCAGGTATGTCG |
| P5 | TVTR10019Phage Hyp-Int_Fw | ACCACAGTGTATCCGGCAAG |
| P6 | TVTR10019Phage Hyp-Int_Rev | AGCATGACGTTGGACGTGTA |
| P7 | TVTR10019Phage Hol-Amid_Fw | TGGCGCAACAACAACATTAC |
| P8 | TVTR10019Phage Hol-Amid_Rev | TGTTGGTTGTTCCTGTGATGA |
| P9 | TVTR10009Phage Int-Hol_Fw | CTCATGACCTGTTCGTGGTC |
| P10 | TVTR10009Phage Int-Hol_Rev | AACGGAAGTAACCGTGATGC |
| P11 | TVTR10009Phage Hyp-Int_Fw | ATTGCCAAGAAGGACATTGC |
| P12 | TVTR10009Phage Hyp-Int_Rev | ATGCGCCATCTTGTATCGAC |
| P13 | TVTR10029Phage Reg-Int_Fw | TCGAGCCATAGGTTGATTGA |
| P14 | TVTR10029Phage Reg-Int_Rev | ATCGACTACCAAGCCGTAGC |
| P15 | TVTR10029Phage Rib_FW | GCATGATCGTCACCAACCTC |
| P16 | TVTR10029Phage Rib_Rev | GCGGTGTAGTCGTTGATGTC |
| P17 | TVTR10029Phage Int-Int_FW | CAAGAATTCTGCGTGTCGTC |
| P18 | TVTR10029Phage Int-Int_Rev | TTGACGGTGTCCTGACTGAG |
| P19 | TVTR10029Phage Int-Hol_FW | GCATAGCAGTGTGGAGACCA |
| P20 | TVTR10029Phage Int-Hol_Rev | AGGAGGCATGATGGATTACG |
| P21 | TVTR10029Phage Hyp-Lys_FW | GACTCTCCAGCAGCTTAACG |
| P22 | TVTR10029Phage Hyp-Lys_Rev | GTAGTGGACGCCGAACATTC |
| P23 | TVTR10029Phage Sugar_FW | GTCGCTCCGGTTAATCTGTG |
| P24 | TVTR10029Phage Sugar_Rev | GAATACAAGCAGCCGACCG |
| P25 | TVTR10035Phage Reg-Int_FW | TACAACAATTCAGCCGAAGC |
| P26 | TVTR10035Phage Reg-Int_Rev | ATCGCGTTGGATAAGGTGAC |
| P27 | TVTR10035Phage Tail_FW | CATTGGTGTGGTTCTTCACG |
| P28 | TVTR10035Phage Tail_Rev | CTCTTCCTCGCGTAGTCGTC |
| P29 | TVTR10035Phage Int-Hol_FW | CCGTGTAGGTGCCTATCCAT |
| P30 | TVTR10035Phage Int-Hol_Rev | TGCCTAACCGAGCCTATGAC |
| P31 | TVTR10035Phage Protein_FW | TGCCAGGCAATACGGTAGAC |
| P32 | TVTR10035Phage Protein_Rev | GTCTCCTCCAGCATTCGTTC |
