# Supplementary figures and images for: Maternal inheritance of bifidobacterial communities and bifidophages in infants through vertical transmission
Source: Microbiome. 2017 Jun 26;5:66. doi: 10.1186/s40168-017-0282-6 (PMC5485682; doi:10.1186/s40168-017-0282-6)

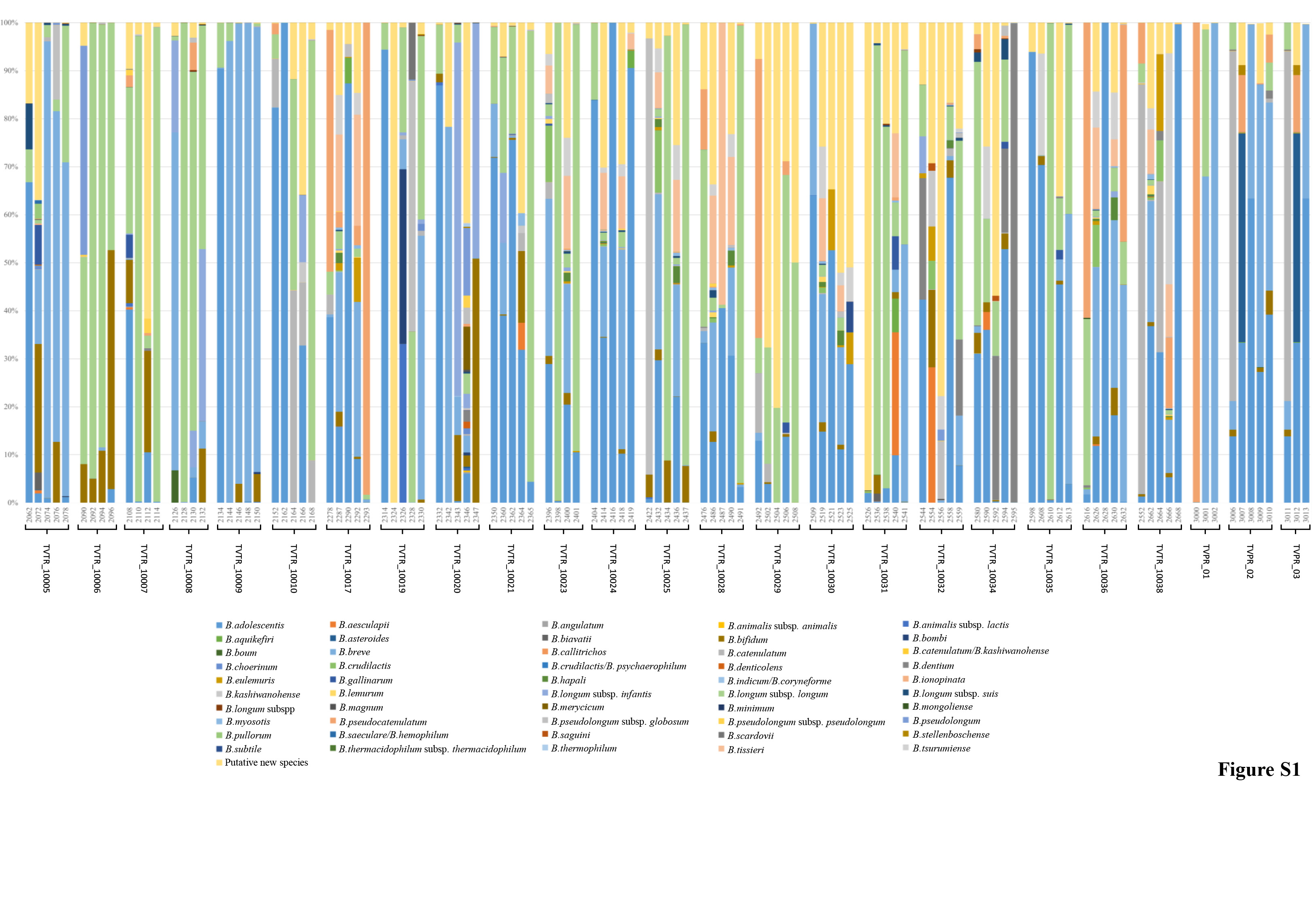

Supplement: Supplementary file 2 — Figure S1. ITS-based OTUs shared between mother-milk-infant sample sets. The bar plot represents the percentage of the total bifidobacterial population found in mother-milk, mother-infant, milk-infant and mother-milk-infant samples. (JPG 1306 kb) [file 40168_2017_282_MOESM2_ESM.jpg]

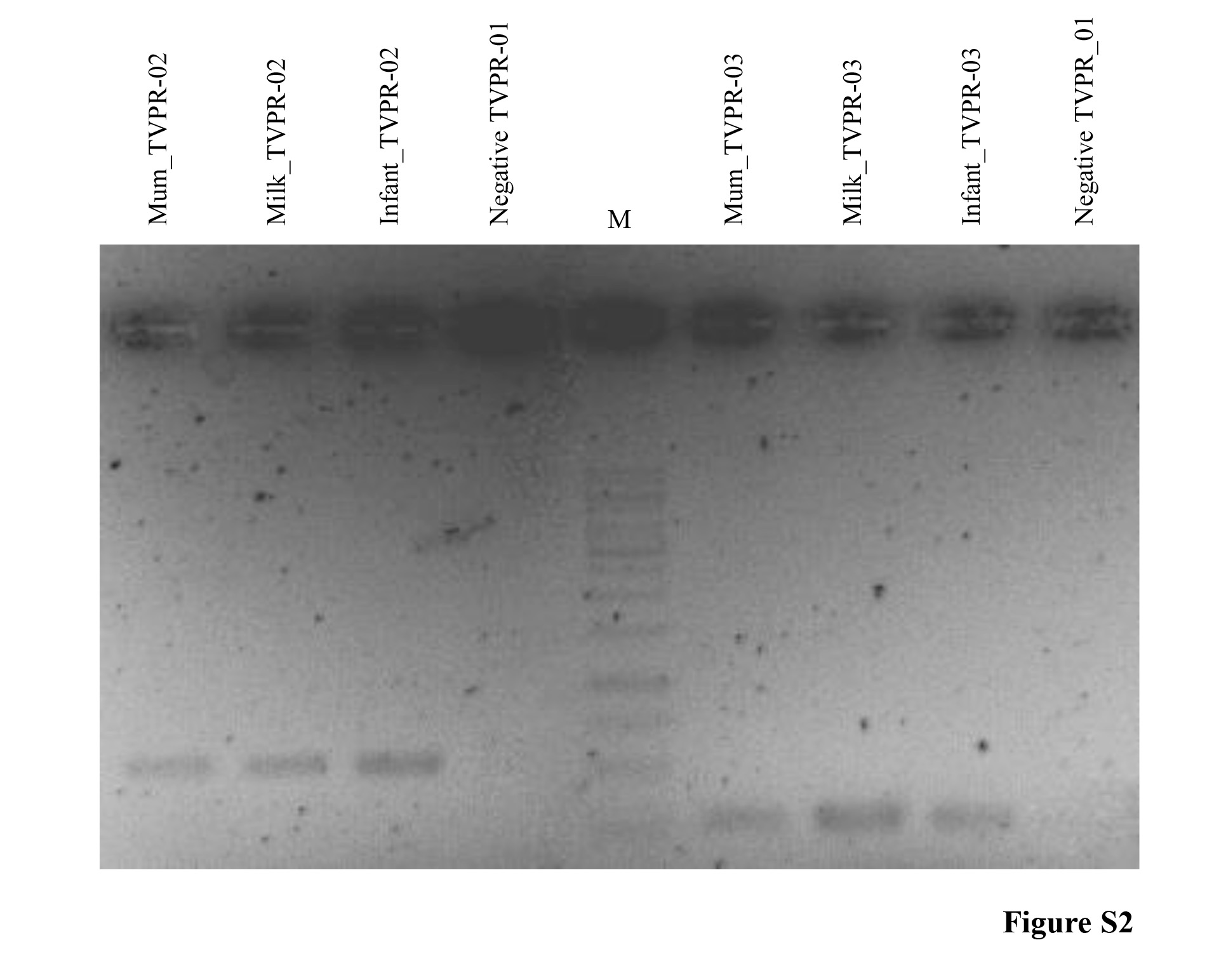

Supplement: Supplementary file 3 — Figure S2. Evaluation of presence of TVPR-02 and TVPR-03 microbiome-reconstructed genomes in fecal samples of the infants and mothers as well as in milk samples. Primer sequences are reported in Additional file 1: Table S2. (JPG 235 kb) [file 40168_2017_282_MOESM3_ESM.jpg]

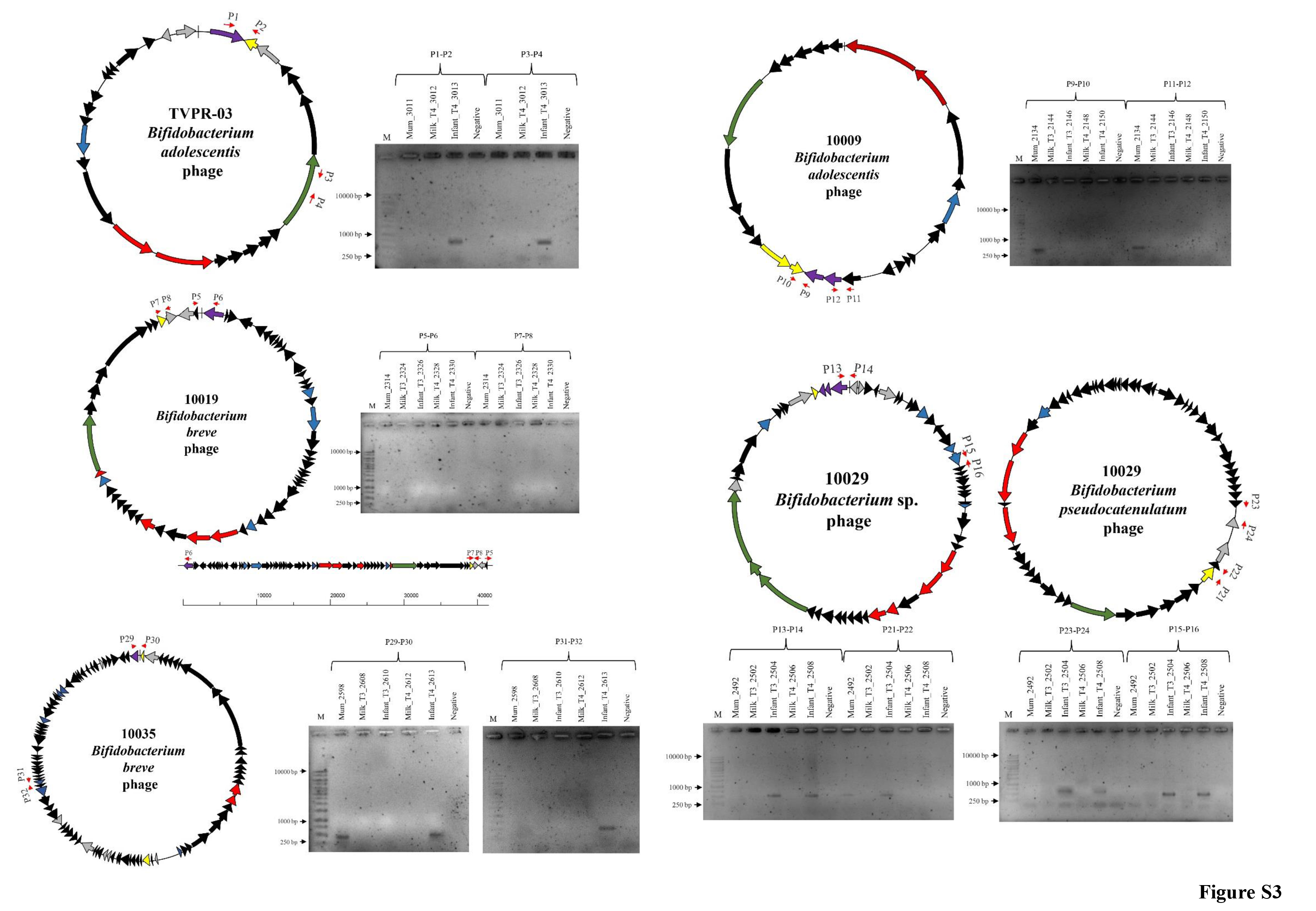

Supplement: Supplementary file 4 — Figure S3. Identified bifidophage/bifidoprophage within the mother’s and infant’s metagenomic samples. Genomic maps of phages recall their modular genomic structure indicated by different patterns, which specifies their predicted function (violet: lysogeny module; blue: DNA replication; red: DNA packaging and head; green: tail and tail fiber; yellow: lysis module; black arrows: hypothetical protein; grey arrows: similar to bacterial protein). For each phage is reported the presence within the mother’s and infant’s metagenomic samples through PCR analysis. Primer sequences are reported in Additional file 1: Table S5. (JPG 1173 kb) [file 40168_2017_282_MOESM4_ESM.jpg]
